# Scanning X-ray strain microscopy of inhomogeneously strained Ge micro-bridges

**DOI:** 10.1107/S1600577513025459

**Published:** 2013-11-02

**Authors:** Tanja Etzelstorfer, Martin J. Süess, Gustav L. Schiefler, Vincent L. R. Jacques, Dina Carbone, Daniel Chrastina, Giovanni Isella, Ralph Spolenak, Julian Stangl, Hans Sigg, Ana Diaz

**Affiliations:** a Institute of Semiconductor and Solid State Physics, Johannes Kepler University Linz, Altenbergerstrasse 69, 4040 Linz, Austria; b Electron Microscopy, ETH Zurich, Wolfgang-Pauli-Strasse 16, 8093 Zurich, Switzerland; c Paul Scherrer Institute, 5232 Villigen PSI, Switzerland; d Laboratory for Nanometallurgy, Department of Materials, ETH Zurich, Wolfgang-Pauli-Strasse 10, 8093 Zurich, Switzerland; e European Synchrotron Radiation Facility, 6 rue Jules Horowitz, BP 220, 38043 Grenoble, France; f L-NESS, Dipartimento di Fisica del Politecnico di Milano, Polo di Como, 22100 Como, Italy

**Keywords:** X-ray diffraction, local probe X-ray diffraction, strain

## Abstract

Strained semiconductors are ubiquitous in microelectronics and microelectromechanical systems, where high local stress levels can either be detrimental for their integrity or enhance their performance. Consequently, local probes for elastic strain are essential in analyzing such devices. Here, a scanning X-ray sub-microprobe experiment for the direct measurement of deformation over large areas in single-crystal thin films with a spatial resolution close to the focused X-ray beam size is presented. By scanning regions of interest of several tens of micrometers at different rocking angles of the sample in the vicinity of two Bragg reflections, reciprocal space is effectively mapped in three dimensions at each scanning position, obtaining the bending, as well as the in-plane and out-of-plane strain components. Highly strained large-area Ge structures with applications in optoelectronics are used to demonstrate the potential of this technique and the results are compared with finite-element-method models for validation.

## Abstract 2

A scanning X-ray strain microscopy technique using a micro-focused beam is demonstrated.

## 1. Introduction

Strain and the thereby induced bending have become important morphological properties of micro- and nano-sized semiconductor components and often have critical impact on the performance of microelectronic (Viktor, 2011 ▶), photovoltaic (Wu *et al.*, 2009 ▶), optoelectronic (Liu *et al.*, 2007 ▶) and microelectromechanical (Serry *et al.*, 1998 ▶) systems. In order to make theoretical predictions about the influence of the strain distribution on other material properties, the deformation of the crystal (*i.e.* lattice strain and bending) needs to be resolved locally. While uniform strain distributions over large areas, such as pseudomorphically grown layer stacks, or averaged periodically strained structures, can be measured easily by X-ray diffraction using a laboratory source, individual strained microstructures and nanostructures require more sophisticated methods in order to analyze their possibly non-uniform strain distribution. The same is valid for free-standing and suspended structures that are often used in micromechanical systems. Those are subject to bending and strain when actuated, which generally is only evaluated by visual methods, such as digital image correlation.

A multitude of methods to study strain is available (Wolf *et al.*, 2003 ▶). Frequently micro-Raman spectroscopy with sub-micrometer resolution is performed non-destructively in a simple laboratory environment and provides an indirect measurement of the strain (*e.g.* Moutanabbir *et al.*, 2010 ▶). However, quantitative data evaluation relies on phenomenological constants (Anastassakis *et al.*, 1970 ▶), and often on simulations as well as the decoupling of local laser heating from strain. Electron backscatter diffraction can deliver high spatial resolution, but, similarly to Raman, it lacks penetration depth, only providing relative values of the strain with a limited strain sensitivity (Villert *et al.*, 2009 ▶). Transmission electron microscopy can be used to determine strain in nanostructures, sometimes with a spatial resolution down to the nanometer scale (Hüe *et al.*, 2008 ▶), but requires an invasive sample preparation, which can cause strain relaxation in the region of interest.

In contrast to the mentioned methods, where the probe depth is wavelength dependent and can be limited to 10–100 nm, X-rays allow structures to be probed to a depth of several tens of micrometers. The main advantage of X-rays compared with other strain probes lies in the possibility of accessing the entire specimen directly with little preparation and in a non-invasive manner, even when systems in operation with a metal or oxide coating are studied. Additionally, X-ray diffraction provides an accurate and direct measurement of the lattice spacing in crystalline samples based on the positions of Bragg reflections in reciprocal space.

X-ray microbeam Laue diffraction is a powerful method for mapping the full strain tensor of single-crystal and polycrystalline materials with sub-micrometer three-dimensional spatial resolution (Larson *et al.*, 2002 ▶). In this technique a white beam focused by Kirkpatrick–Baez mirrors is used to scan the sample, and Laue diffraction patterns are then recorded with a two-dimensional detector and fitted to provide the full strain tensor of the probed part of the crystal.

An alternative approach to map strain in thin films is to use focused monochromatic radiation at certain Bragg reflections and scan the angle around the Bragg peaks at each sample position, resulting in a local strain value that is averaged along the beam path (Murray *et al.*, 2005 ▶; Chrastina *et al.*, 2012 ▶). As an advantage with respect to Laue microdiffraction, the use of monochromatic radiation has the potential of achieving higher lateral resolution making use of state-of-the-art diffractive and refractive X-ray optics, which provide beams of a few tens of nanometers (Vila-Comamala *et al.*, 2011 ▶; Schroer *et al.*, 2005 ▶). Additionally, it avoids complicated analysis of Laue diffraction patterns (Chung & Ice, 1999 ▶). However, a point-by-point measurement of the strain is often time consuming, which impedes the investigation of large areas.

In this study, a practical implementation of scanning X-ray microdiffraction is presented for the determination of strain and bending in crystalline thin films, using a combination of a monochromatic X-ray sub-micrometer probe and a state-of-the-art fast-readout pixel X-ray detector. In its acquisition scheme the sample is continuously scanned with respect to the X-ray beam, while the detector progressively acquires images. This scheme is performed at different incident angles of the beam close to a Bragg reflection, thereby mapping a part of reciprocal space in three dimensions at each scanning position in real space. As is shown in this work, this leads to a direct measurement of the displacement field and the lattice curvature as a function of the sample position. The final data are obtained without the need of fitting the recorded Bragg peaks and thus offer easy and fast mapping possibilities. In contrast to diffraction experiments with a large beam, this approach is model independent and does not rely on the fitting of theoretical scattering intensities to measured ones by optimizing a structural model of the sample at all. The practicability of the method is demonstrated by mapping the in-plane and out-of-plane strain components together with the lattice curvature of a Ge bridge structure with applications in optoelectronics, covering a field of view of 90 µm × 34 µm with a step size of 1 µm.

The sample description together with the experimental setup and the data acquisition and reduction is provided in §2. Maps of the obtained bending and strain components are shown in §3 and compared with finite-element-method (FEM) calculations for validation. This is followed by a brief discussion of the applicability of the method in §4, and §5 summarizes the work and provides perspectives for future applications.

## 2. X-ray microdiffraction experiment and simulation

### 2.1. Sample

**Figure 1:**
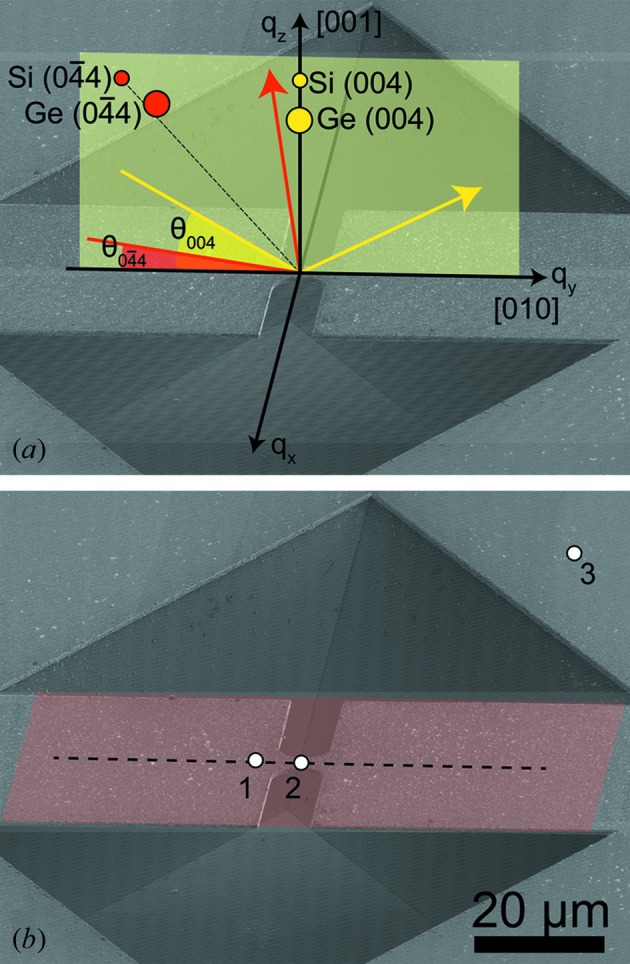
(*a*) Scanning electron microscopy (SEM) image of a typical Ge micro-bridge used in the experiments with a sketch of the scattering geometry indicating the incoming and outgoing wavevectors for the two probed Bragg reflections. (*b*) The same bridge structure as that shown in (*a*) with a colored plane indicating the region scanned along the sample surface and three points where measurements described in the text have been performed.

Experiments were conducted on strain-enhanced micro-bridges fabricated from a Ge layer on a Si substrate (see Fig. 1 ▶). Bulk Ge has an indirect band gap with a local conduction-band minimum at the Γ-point. When enough tensile strain is applied, the energy of the direct band gap is eventually lowered below the indirect conduction-band minimum at the L-point (Vogl *et al.*, 1993 ▶). A true direct band gap is expected for uniaxial strain above 4% along the [100] direction (Aldaghri *et al.*, 2012 ▶). In the sample presented in this work, peak strains occur in the center of a micro-bridge constriction and the achieved strain enhancement depends on cross-section variations within the structure. This concept is used in mechanics and material science for testing purposes (Serope & Steven, 2010 ▶; Gravier *et al.*, 2009 ▶), but was also demonstrated as a method of strain enhancement and thus band-gap engineering for semiconductors (Minamisawa *et al.*, 2012 ▶; Süess *et al.*, 2013 ▶).

For the sample fabrication a 2.1 µm-thick Ge layer was grown directly on a (001) Si substrate by low-energy plasma-enhanced chemical vapor deposition (Isella *et al.*, 2004 ▶). Owing to the thermal mismatch in expansion coefficients of Si and Ge, a biaxial tensile in-plane strain within the Ge layer remains after cooling the sample down to room temperature.

The dumbbell-shaped micro-bridges were fabricated from 2.2 cm × 2.2 cm Ge on Si chips using e-beam lithography, reactive ion-etching and wet chemical etching. A 60 nm Cr layer and a 300 nm spin-coated polymethyl methacrylate (PMMA) layer were used as reactive ion etching and e-beam lithography masks, respectively. E-beam patterns with a dumbbell shape were written using a VISTEC e-beam system with 800 µC cm^−2^ dose, at a current between 20 and 150 nA. After developing, the PMMA pattern was transferred into the Cr layer via a Cl_2_ plasma. The samples were then reactive ion-etched using a CHF_3_/O_2_/SF_6_ plasma (40/5/2). Thereafter, the remaining PMMA and the Cr mask were removed by a removal agent [5 g of potassium hydroxide (KOH) and 13 g of potassium ferricyanide dissolved in 100 ml of deionized water]. In order to protect the back of the sample during wet etching a KOH resist was deposited. The samples were wet-etched in a 20 wt% KOH solution at 346 K in order to completely remove the Si under the patterned Ge layer, which resulted in a bridge structure where both the constriction and the wider parts are suspended (see Fig. 1 ▶). Post-treatment included surface cleaning by dipping in hot water (2 min at 346 K), a water bath (12 h at 298 K) and subsequent isopropyl alcohol evaporation.

### 2.2. Experimental setup

X-ray diffraction measurements were conducted at the ID01 beamline at the European Synchrotron Radiation Facility in Grenoble, France. A monochromatic beam of 8 keV energy was focused by a Fresnel zone plate (FZP) of 200 µm diameter and 70 nm outer-most zone width, having a focal distance of 90.4 mm and a focal depth of about 100 µm, resulting in a divergence of 0.12° (Gorelick *et al.*, 2011 ▶). A central beam stop and an order-sorting aperture were placed before and after the FZP, respectively, in order to reduce the intensity of the transmitted X-ray beam and to remove non-relevant diffraction orders. The size of the focal spot was determined to be 630 nm × 230 nm [horizontal × vertical, full width at half-maximum (FWHM)]. The sample was placed at the focal plane of the FZP on a diffractometer equipped with an *xyz* scanning piezoelectric stage, with a lateral stroke of 100 µm and a resolution of 2 nm. The dedicated nanobeam instrument at ID01 ensures that unintended sample displacements during angular movements stay below 100 nm for 2° rotation around any Bragg peak. A Maxipix detector with 516 × 516 pixels and 55 µm pixel size (Ponchut *et al.*, 2011 ▶) was used at a distance of 774 mm from the sample.

**Figure 2:**
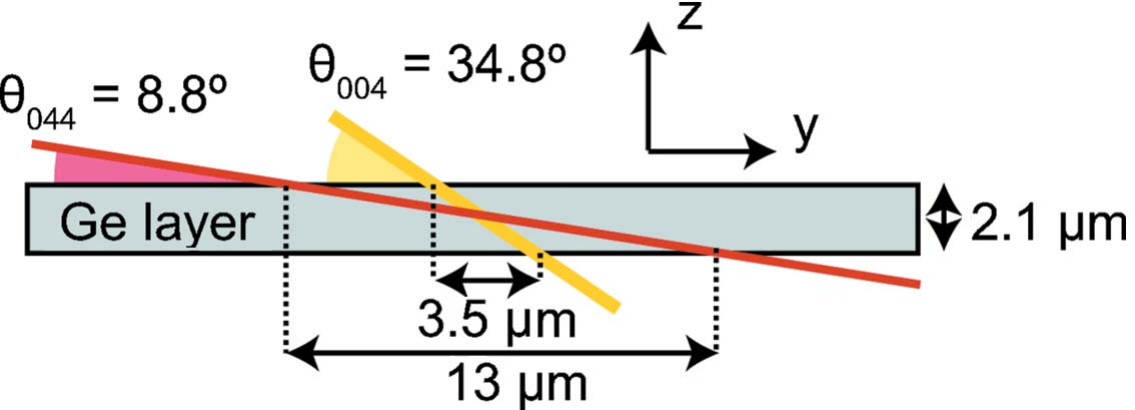
Sketch of the X-ray beam penetration in the sample for both the (004) (yellow) and the 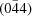 (red) reflections.

In Fig. 1(*a*) ▶ a sketch of the scattering geometry used in the experiment is shown. A local coordinate system with the *x* and *y* directions in the sample plane and the *z* direction perpendicular to the plane is used. The sample was mounted with the [010] crystallographic direction along the *y* direction. For measurements the sample was tilted close to the nominal Si (004) Bragg reflection at 34.80°, and the detector arm was moved to twice this value. Owing to the non-perpendicular incidence angle of the beam with respect to the sample surface, the effective beam size on the sample surface was 400 nm along the *y*-direction. In order to access the 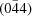 Bragg reflection of Si, the sample was tilted to an angle of 8.81° with respect to the incoming beam and the detector arm was tilted to an angle of 107.98°, resulting in a footprint of the beam on the sample surface of 1500 nm along the *y*-direction. The resulting penetration length of the beam through the 2.1 µm-thick Ge layer measures several micrometers along the *y*-direction, being especially large in the case of the 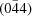 reflection, as shown schematically in Fig. 2 ▶. This problem can be overcome by changing from low-incidence to high-incidence diffraction geometry, *i.e.* changing from the 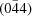 to the (044) reflection. Another possibility would be to use a higher energy and thus different reflections with almost perpendicular incidence. In the current study, spatial constraints of the beamline setup prevented the experiment from being conducted in such a configuration. Nevertheless, it should be pointed out that the outlined scheme could easily be applied to smaller beams, if available, which could improve the resolution to the sub-hundred nanometer regime.

### 2.3. Data acquisition

**Figure 3:**
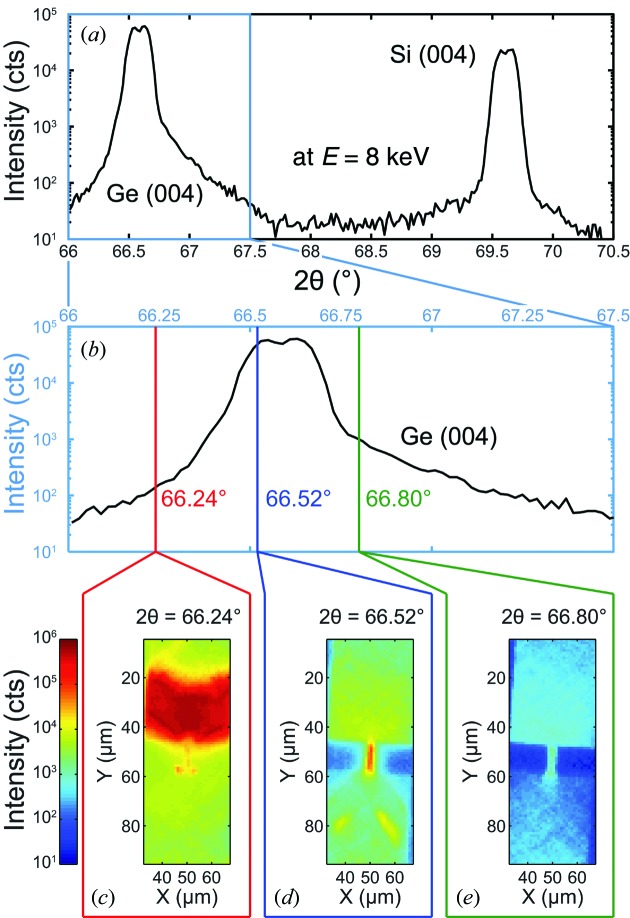
Intensity map at different positions on the Ge (004) reflection. (*a*) Radial scan across the Ge and Si (004) reflections recorded at position 3 displayed in Fig. 1(*b*) ▶. (*b*) Detail around the Ge (004) reflection. (*c*)–(*e*) Meshes recorded at different Bragg angles as indicated in (*b*). Note: the peak shape is a result of the convolution with the hollow-cone-shaped primary beam caused by the FZP.

For each probed Bragg reflection first a radial scan (in which the direction of the scattering vector remains constant while its length is changed) at a position on the sample away from the bridge structure [see position 3 in Fig. 1(*b*) ▶] was performed. As shown in Fig. 3(*a*) ▶, this scan served to determine the exact position of the Si peak, which was later used as a reference for the strain measurements. A biaxial tensile strain of 0.18% was retrieved from the Ge peak position in these scans.

In order to measure the out-of-plane strain component and the bending, the sample was laterally scanned in real space, covering a total area of 90 µm × 34 µm with a step size of 1 µm at 31 angular positions within a scan range of 0.3° around the Ge reflection, resulting in a step size of 0.01°, well below the divergence of the focused beam.

In order to speed up the measurements, a continuous scan modality was implemented (similar to, for example, Menzel *et al.*, 2010 ▶), in which for each line of the mesh scan the piezoelectric stage continuously moves along one direction and periodically triggers detection. During this time a buffer of images is stored without feedback to the control system. These measurements were performed at both the (004) and the 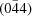 reflections, collecting a total of 31 real-space two-dimensional maps per Bragg peak, yielding a total number of 98735 detector frames per reflection. The total recording time of the map per reflection was approximately 4 h, resulting in an average collection rate of about seven frames per second.

In Figs. 3(*c*)–3(*e*) ▶ three of the collected two-dimensional intensity maps are shown as examples, which were recorded at the three angular positions marked in Fig. 3(*b*) ▶. The images are obtained using the integrated intensity of the whole detector area (approximately 2° in each direction) at each scanning position. This provides diffraction contrast and allows for the identification of the sample position with respect to the focused beam (Mocuta *et al.*, 2008 ▶; Hrauda *et al.*, 2011 ▶). In order to reconstruct the full three-dimensional reciprocal space map (RSM) at each scanning position, a full set of maps at different incidence angles has to be recorded. These maps contain detailed information on the local strain and the bending of the suspended bridge.

### 2.4. Data reduction

**Figure 4:**
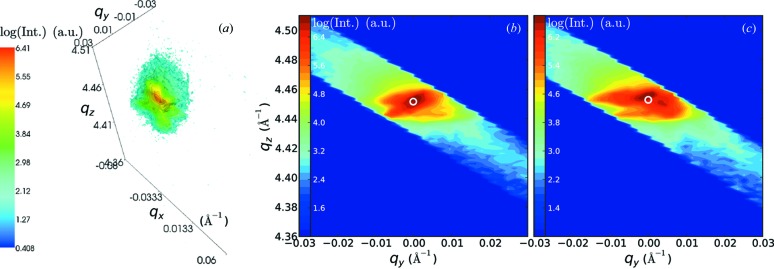
(*a*) Reconstruction of a (004) Ge Bragg peak in three-dimensional reciprocal space measured at position 1 indicated in Fig. 1(*b*) ▶. (*b*) and (*c*) Projections onto the scattering plane of three-dimensional reciprocal space at positions 1 and 2 indicated in Fig. 1(*b*) ▶, respectively.

The obtained detector frames around the two different Bragg peaks of a full set of maps are reconstructed into three-dimensional RSMs by translating angular coordinates to reciprocal-space coordinates (Lazzari, 2002 ▶; Kriegner, 2013 ▶), 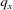, 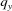 and 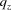, as shown in Fig. 4(*a*) ▶. The average Bragg position is obtained by computing the center of mass (COM) of the Bragg peak,
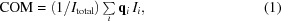
where the sum runs over all reciprocal space points 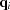 with the corresponding scattering intensities 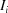. This data treatment is inspired by scanning transmission X-ray microscopy with pixelated detectors (Menzel *et al.*, 2010 ▶) where the deflection of the beam is used to image objects in transmission with differential phase contrast. In the present work the COM provides a direct measurement of strain and bending in the sample and it can be determined with a much higher precision than the beam divergence. Tilt corrections, more precisely the deviation of the incidence angle θ from half of the detector angle (defined between the incidence and scattered beam, *cf*. Fig. 1 ▶), are calculated from the COM of the (004) maps as well and then applied to the 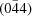 maps in order to correct angular offsets.

Figs. 4(*b*) and 4(*c*) ▶ show (004) maps obtained at two different positions on the bridge [*cf*. white dots in Fig. 1(*b*) ▶] projected onto the scattering plane. The in-plane and out-of-plane lattice parameter are retrieved from the COM (indicated by white circles), which can be used to calculate the strain components 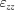 and 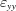, respectively, averaged over the small illuminated area, as well as the bending in the *x*- and *y*-direction. This analysis is performed at each real-space scanning point, effectively obtaining two-dimensional strain and bending maps of the sample. For both the (004) and the 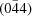 reflections, movies showing the Bragg peak movement as a function of the beam position along a line through the center of the bridge can be found in the supporting information (SI)^1^.

### 2.5. FEM simulations

**Figure 5:**
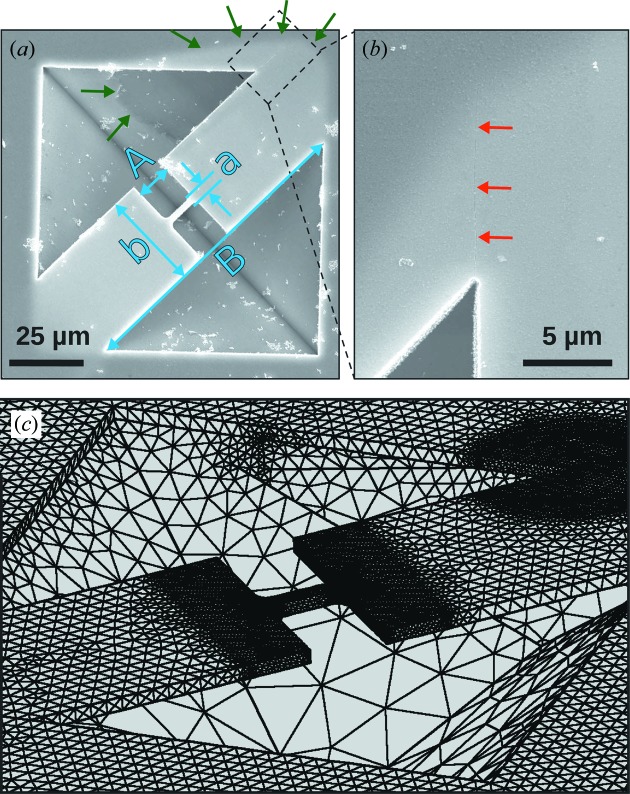
(*a*) Detailed SEM micrograph of the fabricated bridge structure revealing asymmetric etching effects, marked by green arrows. (*b*) SEM micrograph revealing cracks in the investigated structure, highlighted by red arrows. (*c*) Mesh of the FEM model used for calculation, which includes the defects displayed in (*a*) and (*b*).

To verify our results, FEM calculations were conducted with the software package *COMSOL Multiphysics 4.3*, using the linear elastics module. The dimensions of the structure, the stiffness tensor entries as well as the densities of Si and Ge served as model input parameters (Wortman & Evans, 1965 ▶). The simulation included a 2.1 µm Ge layer on top of a Si substrate and an initial strain state with 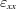 = 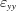 = 0.18% and 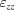 = −0.12%, which was patterned with the shape of the measured bridge. The orientation was chosen with the bridge direction along [010], as was the case in the real sample. Asymmetric etching effects [Fig. 5(*a*) ▶] and cracks [Fig. 5(*b*) ▶] found through scanning electron microscopy (SEM) inspection of the sample were incorporated into the FEM model Fig. 5(*c*) ▶].

The effect of beam size and penetration depth was investigated by convolving the simulated strain distribution 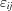 with a beam distribution function at each pixel,
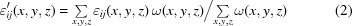
with
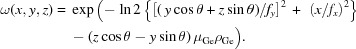
The function 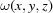 represents a simulated illumination function in three dimensions, with a two-dimensional Gaussian profile in the plane perpendicular to the direction of the incoming beam and with an attenuation along the beam path through the material, similar to that used by Diaz *et al.* (2010 ▶). Here, 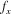 and 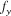 are the FWHM of the beam in the plane perpendicular to the beam propagation direction, 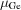 = 68.9 cm^2^ g^−1^ and 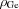 = 5.323 g cm^−3^ are the mass attenuation coefficient of Ge at 8 keV and the density of Ge (Levinshtein *et al.*, 2001 ▶), respectively. In order to account for the incoming beam direction the coordinate system was rotated around the *y*-axis of the coordinate system shown in Fig. 1(*a*) ▶ by the beam incidence angle θ.

## 3. Results

### 3.1. Bending

**Figure 6:**
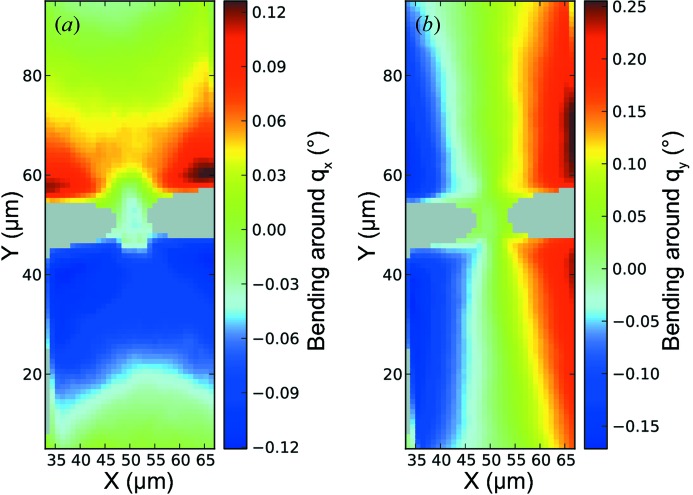
(*a*) and (*b*) Bending around 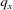 and 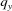, respectively, determined from the (004) Ge Bragg reflection.

Local tilts of the lattice arising from the distortion of the bridge due to strain result in deviations of the COM from zero in the 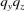
- and the 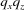-plane obtained from the (004) maps. Evaluating these deviations allows thus the determination of the bending of the bridge with respect to the *x*- and *y*-direction, respectively, and leads to a visualization of the curvature of the bridge. With this approach the bending of the structure can be determined locally with a resolution below a hundredth of a degree, which is shown in Fig. 6 ▶.

### 3.2. Strain mapping

**Figure 7:**
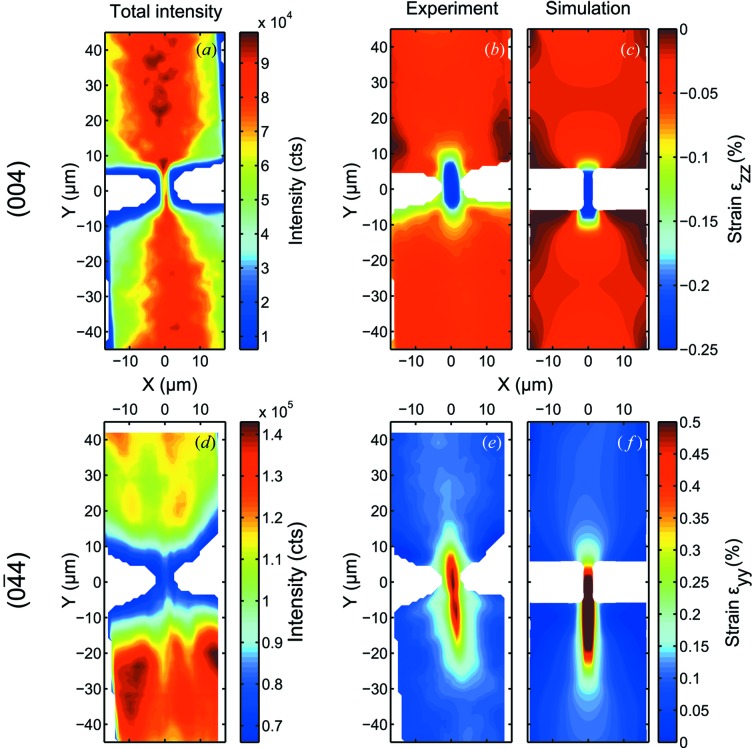
Comparison of measured and calculated strain in an under-etched micro-bridge structure over the region indicated with a colored plane in Fig. 1(*b*) ▶. (*a*) Total integrated intensity of the measured (004) Bragg reflection. (*b*) Measured strain along the [001] direction 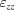 and (*c*) FEM calculation for a structure of identical dimensions. Both results are plotted on the same color scale for the strain values. (*d*) Total integrated intensity of the measured 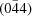 Bragg reflection. (*e*) Measured strain along the [010] direction 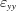 and (*f*) FEM calculation. The slight asymmetry with respect to the *x*-direction observed in (*c*) and (*f*) arises from asymmetric cracks considered in the FEM model according to defects observed in SEM inspections of the sample. The beam impinges on the sample along the positive *y*-direction, as indicated in Fig. 1(*a*) ▶.

In Figs. 7(*a*) and 7(*d*) ▶ maps of the integrated intensity of the entire three-dimensional RSM of the two Bragg peaks obtained as described in §2.4 for the (004) and the 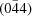 reflections, respectively, are shown. These maps clearly reveal the shape and the position of the bridge structure, which is under-etched and suspended in air. For clarity, intensities below a certain value are represented in white.

A map of the measured out-of-plane strain tensor component 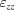 obtained from analysis of the (004) Bragg reflection is shown in Fig. 7(*b*) ▶, and a FEM calculation after convolution with the illumination function is shown in Fig. 7(*c*) ▶.

Measurements and calculations agree very well, revealing a large tensile strain in the center constriction and a smaller strain in the wider part of the bridge structure. For the in-plane tensor component 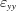, derived from the 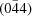 Bragg reflection, a good agreement between measured and simulated strain data was found as well, shown in Figs. 7(*e*) and 7(*f*) ▶, respectively. In this case the asymmetry caused by the interaction volume of the beam is more pronounced due to the smaller incidence angle for the 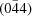 reflection, as shown in Fig. 2 ▶.

### 3.3. Quantitative comparison with FEM calculations

**Figure 8:**
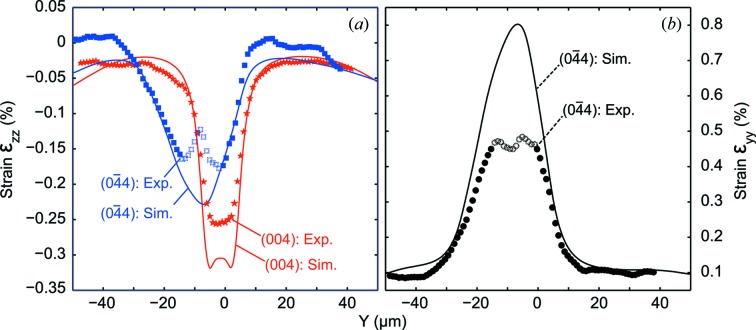
Comparison of measured and calculated strain of the micro-bridge structure. Detail along the dashed line in Fig. 1(*b*) ▶. The open symbols correspond to scan points where it was not possible to reliably determine the COM for reasons discussed in the text.

Fig. 8 ▶ shows a line scan at 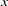 = 0 [corresponding to the dashed line in Fig. 1(*b*) ▶] across the 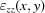 distribution [retrieved from both the (004) and 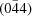 Bragg peaks] and along the 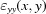 distribution [retrieved from the 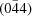 Bragg peak]. In each case, both the measured and calculated strains are shown with symbols and continuous lines, respectively.

In principle, the strain values for 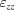, shown in Fig. 8(*a*) ▶, should be identical for both reflections. But the inhomogeneities observed already in the intensity distributions, which are accentuated when comparing line scans, are due to several effects: (i) The interaction volume of the beam is not exactly the same for both reflections due to the geometry of the structure and the angle of the incoming beam (see Fig. 2 ▶). The lower incidence angle at the 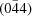 reflection results in a lower lateral resolution compared with the (004) reflection. (ii) Unfortunately, at the 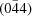 reflection not all peaks were fully recorded. The signal moved out of the recorded angular range when exceptionally large peak shifts occurred due to strain or bending of the sample (see movies in the SI). (iii) The 516 × 516 pixel detector actually consisted of four 256 × 256 pixel chips, separated by 4 pixel-wide stripes, which did not record data. It is likely that for some detector frames parts of the intensity fell within this blank space, and thus part of the total intensity is lost.

Nevertheless, the convolution of the calculated 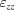 with a beam in the (004) geometry, indicated by a continuous red line in Fig. 8(*a*) ▶, is only slightly shifted with respect to the center of the bridge, and reaches values up to about −0.32%, similar to the unconvolved FEM calculation (not shown). Consequently, although the 3.5 µm penetration along the *y*-direction in this geometry determines the effective resolution along this direction, it should not affect the quantitative strain values measured in the center of the bridge. The measured 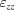 in the middle of the bridge is smaller than expected, reaching values of up to −0.25% only, probably as a consequence of defects in the microstructure which were not taken into account in the FEM model.

The calculated strain convoluted with the beam in the 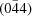 geometry, indicated by the continuous blue line, has a significant shift towards negative *y* values and is considerably smeared out as a consequence of the grazing θ, which in this case limits the resolution to about 13 µm. Similarly, the calculated 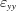 values indicated in Fig. 8(*b*) ▶ with a continuous black line are shifted and smeared considerably. The strain components measured with the 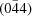 reflection in the middle of the bridge have artificially lower values originating from peak splitting and shifting out of the detector range. The reason for the peak splitting is again the grazing geometry, where especially near the center of the bridge parts of the bridge with completely separated strain values were probed. This artifact is portrayed in the movie of the peak movement along a line scan for the 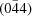 reflection (see movie 2 in the SI). Measurement points where the peak has shifted out of the measurement range have been indicated with empty symbols in Fig. 8 ▶.

## 4. Discussion

The method reported above constitutes a fast and practical way of mapping curvature and strain on a sample of many tens of micrometers in lateral size with sub-micrometer resolution, ultimately limited by the footprint of the beam on the sample. Since nano-focused beams are becoming more and more easily available, the discussed implementation could be realised at beamlines where a micrometer-range sample translation together with a fast readout of the two-dimensional detector is available. The information gained in the experiment are bending and strain maps of the whole area, although averaged along the direction of the incoming beam.

The diffraction patterns shown in this work (see Fig. 4 ▶) show a large number of features which were disregarded, but which could be further exploited by comparison with patterns obtained from FEM calculations in combination with scattering simulations. Even though the reciprocal-space resolution is limited by the rather large divergence of the beam, comparative studies could reveal more complete structural and strain analysis on the local level, as has been done, for example, by Mocuta *et al.* (2008 ▶).

In the proposed scheme, reciprocal-space details are compromised for the sake of measurement speed, optimizing acquisition time in order to obtain fast curvature and strain maps of the sample. Furthermore, by omitting the reciprocal-space details, the continuous acquisition scheme together with the short read-out times of the Maxipix detector would allow even faster scans. The RSMs recorded then would not reveal detailed intensity distributions, but would still be good enough for an averaged strain measurement by computing the COM. We envision that such fast scans could be implemented in diffraction synchrotron beamlines together with appropriate analysis tools, such as those presented here, allowing a quick curvature and strain map determination within a few minutes. Such overview scans could be then used to find regions of interest on the sample for further detailed analysis, *e.g.* Bragg ptychography (Godard *et al.*, 2011 ▶; Hruszkewycz *et al.*, 2012 ▶). For this method, coherent illumination and extremely high stability are required. For practical reasons, measuring a field of view as large as measured in this experiment is currently out of reach. However, the presented scheme would be an ideal tool for pre-selecting a specific region of interest, on which Bragg ptychography could be performed to retrieve a high-resolution three-dimensional strain map.

## 5. Conclusion and outlook

An implementation for fast-scanning X-ray diffraction microscopy in large areas is reported and applied for the determination of the curvature and strain in a 2 µm-thick Ge micro-bridge as a benchmark.

(i) Iteratively, a piezo-stage scans a sample under a focused X-ray beam and a diffractometer is moved to a different angular position, effectively scanning an angular range around the Bragg reflection. In this way two-dimensional maps in real space at different incidence angles can be recorded quickly.

(ii) By translating angular space into reciprocal space the recorded detector images are reconstructed into three-dimensional RSMs for each direct-space scan position.

(iii) From the three-dimensional RSMs the average Bragg peak position is retrieved by center-of-mass calculation, which is then used for determination of bending, local tilt and strain.

(iv) In order to obtain more than one strain tensor component, more than one Bragg peak needs to be measured. Additionally, since the position of the peaks may be affected by the bending of the whole layer, information retrieved from a symmetric peak needs to be used to correct the position of any asymmetric peak.

This is a practical method for probing strain and bending in two-dimensional films where refraction effects due to changes in the sample geometry can be neglected. Despite using a highly focused X-ray beam, the strain sensitivity can be much better than the divergence of the beam by computing the center of mass of the reciprocal-space peak at each scanning position. Apart from providing a direct measurement of curvature and strain, this method can be particularly relevant for the study of buried films or integrated structures, where other methods cannot access strain without destroying the sample. The spatial resolution can be better than that achieved by other methods by using high-resolution X-ray optics, which can currently focus X-rays down to 100 nm or below. We therefore propose this technique as a fast and uncomplicated way for imaging and analyzing strain and curvature in sub-micrometer-patterned crystalline layers such as those used in electronic devices.

## Supplementary Material

Movie 1. DOI: 10.1107/S1600577513025459/fv5011sup1.mp4

Movie 2. DOI: 10.1107/S1600577513025459/fv5011sup2.mp4

## Footnotes

1 Supporting data for this paper are available from the IUCr electronic archives (Reference: FV5011).
